# A psychometric examination of the career construction model of adaptation in working adults and students

**DOI:** 10.3389/fpsyg.2026.1799936

**Published:** 2026-07-27

**Authors:** Yoke Loo Sam, Georgios Christopoulos, Marilyn A. Uy, Shen-Hsing Annabel Chen, Henriette Hendriks, Trevor W. Robbins, Barbara Sahakian, Victoria Leong, Zoe Kourtzi, The CLIC Consortium

**Affiliations:** 1 Centre for Research and Development in Learning, Nanyang Technological University, Singapore, Singapore; 2 Nanyang Business School, Nanyang Technological University, Singapore, Singapore; 3 School of Social Sciences, Nanyang Technological University, Singapore, Singapore; 4 Department of Theoretical and Applied Linguistics, University of Cambridge, Cambridge, United Kingdom; 5 Department of Psychology, University of Cambridge, Cambridge, United Kingdom; 6 Department of Psychiatry, University of Cambridge, Cambridge, United Kingdom

**Keywords:** adaptability, adaptation, adapting, adaptivity, career construction model of adaptation, psychometrics, career adaptability

## Abstract

The Career Construction Model of Adaptation (CCMA) is a widely adopted multidimensional framework that conceptualizes career development through four interconnected dimensions: *adaptivity, adaptability, adapting*, and *adaptation* (“four As”). Despite its theoretical prominence, empirical research on the CCMA remains fragmented, with most studies focusing on individual dimensions or isolated measures rather than testing the model as a coherent latent structure. In Study 1 (*N* = 1,000 working adults), we applied psychometric techniques to examine the shared variances among seventeen established career-related measures aligned with the “four As.” Factor analysis supported a parsimonious four-factor model consistent with the CCMA framework, and subsequent structural path modeling with bootstrapping further supported a serial mediation process in which *adaptivity* (A1) predicted *adaptation* (A4), both directly and indirectly via *adaptability* (A2) and *adapting* (A3). Study 2 (*N* = 323 university students) provided evidence of structural consistency across a sample at a different career stage, with variations attributable to early career characteristics. Our results offer one of the first integrative psychometric evaluations of the CCMA, demonstrating its viability as a multidimensional latent system. These findings contribute to ongoing efforts to clarify the empirical foundations of career construction theory and support future applications of the CCMA in research and practice.

## 1 Introduction

Amidst the challenges stemming from the repercussions of the Coronavirus pandemic and the rise of artificial intelligence (AI), the global economy faces unprecedented volatility, uncertainty, complexity, and ambiguity (VUCA; Barber, 1992), further obscuring the already turbulent job market prospects. In a study conducted by the US Bureau of Labor Statistics, Dell et al. (2020) examined the impact of pandemic-driven technology and AI on the workforce, highlighting increased demand for digital skills, job displacement and transformation, as well as the emergence of new roles centered on training and maintaining AI systems. Goldman Sachs's (2023) projection of 300 million potential job replacements due to AI highlights its substantial effect on the global workforce. Initially perceived as affecting primarily low-skilled workers (Boo, 2023; Fanusie, 2021), the increasing sophistication of AI is beginning to extend its influence into knowledge-worker roles (Hering, 2023). Individuals must develop greater adaptability to navigate the evolving post-pandemic, AI-driven world and respond to unforeseen disruptions throughout their careers. In light of these sweeping changes, it becomes increasingly critical to examine the competencies that enable individuals to proactively manage career transitions and uncertainties. One such competency is career adaptability.

Career adaptability is a meta-competency that empowers individuals to effectively solve unfamiliar, complex problems in vocational tasks, ensuring smooth career development and transitions (Savickas and Porfeli, 2012). Originally introduced by Super and Knasel (1981), career adaptability has since gained widespread recognition as a cornerstone of contemporary career development theory and practice. Expanding on the conceptualization of career adaptability, Savickas (2002, 2013) introduced career construction theory to offer a broader framework for understanding how individuals actively construct their careers in response to the complexities of an evolving world. Unlike deterministic theories such as person-environment fit (e.g., Holland's theory of career choice, Holland, 1959) or lifespan approaches (e.g., Super's developmental self-concept theory, Super, 1985), career construction theory adopts a “contextualist” framework (Hartung and Cadaret, 2017) that highlights the interplay between personal attributes and social conventions in achieving successful career development (Savickas, 2013; Savickas and Porfeli, 2012).

Central to career construction theory is the idea that career outcomes depend on an individual's ability to balance authenticity and social recognition by negotiating their vocational personality with the demands of career changes (Savickas, 2013). Building on this foundation, Savickas and Porfeli (2012) introduced the career construction model of adaptation (CCMA), a multidimensional framework that operationalizes career development through four interconnected dimensions: *adaptivity, adaptability, adapting*, and *adaptation*—collectively known as the “four As” in this paper. *Adaptivity* is a stable trait reflecting an individual's willingness to adapt to workplace changes, especially when established routines no longer suffice (Savickas, 2002, 2013). *Adaptability* refers to the malleable capacity for proactively strategizing and managing career challenges (Savickas and Porfeli, 2012). *Adapting* responses involve behaviors and beliefs used to navigate career demands, such as skill mastery and coping with transitions (Rudolph et al., 2017). *Adaptation* reflects the outcomes of these responses, including job satisfaction and employability (Savickas, 1994).

According to the CCMA, *adaptivity* influences the development of *adaptability*, which enables *adapting* responses that lead to positive *adaptation* outcomes, underscoring the interconnectedness of the “four As” in facilitating successful career development (Savickas and Porfeli, 2012). To reflect this theoretical sequence, a serial mediation model was used to examine the hypothesized causal chain. A meta-analysis conducted by Rudolph et al. (2017) further supports this model by examining associations among the four dimensions across an extensive list of measures, offering a broader perspective on how various career-related constructs interact to influence career outcomes.

### 1.1 Challenges in measuring and validating the career construction model of adaptation

Recent years have seen a marked rise in CCMA-related studies that incorporate all four dimensions of adaptation (e.g., Green et al., 2023; Leung et al., 2022; Merino-Tejedor et al., 2016; Nilforooshan, 2020; Perera and McIlveen, 2017; Savickas et al., 2018; Šverko and Babarović, 2019; Zhou et al., 2016). The inherent breadth of the CCMA enables exploring how various career-related constructs are associated with one another. This broad range of factors contributing to the career construction process demonstrates the model's capability to extend beyond single variables and captures the complexity of individual characteristics shaping career development.

**Figure 1:**
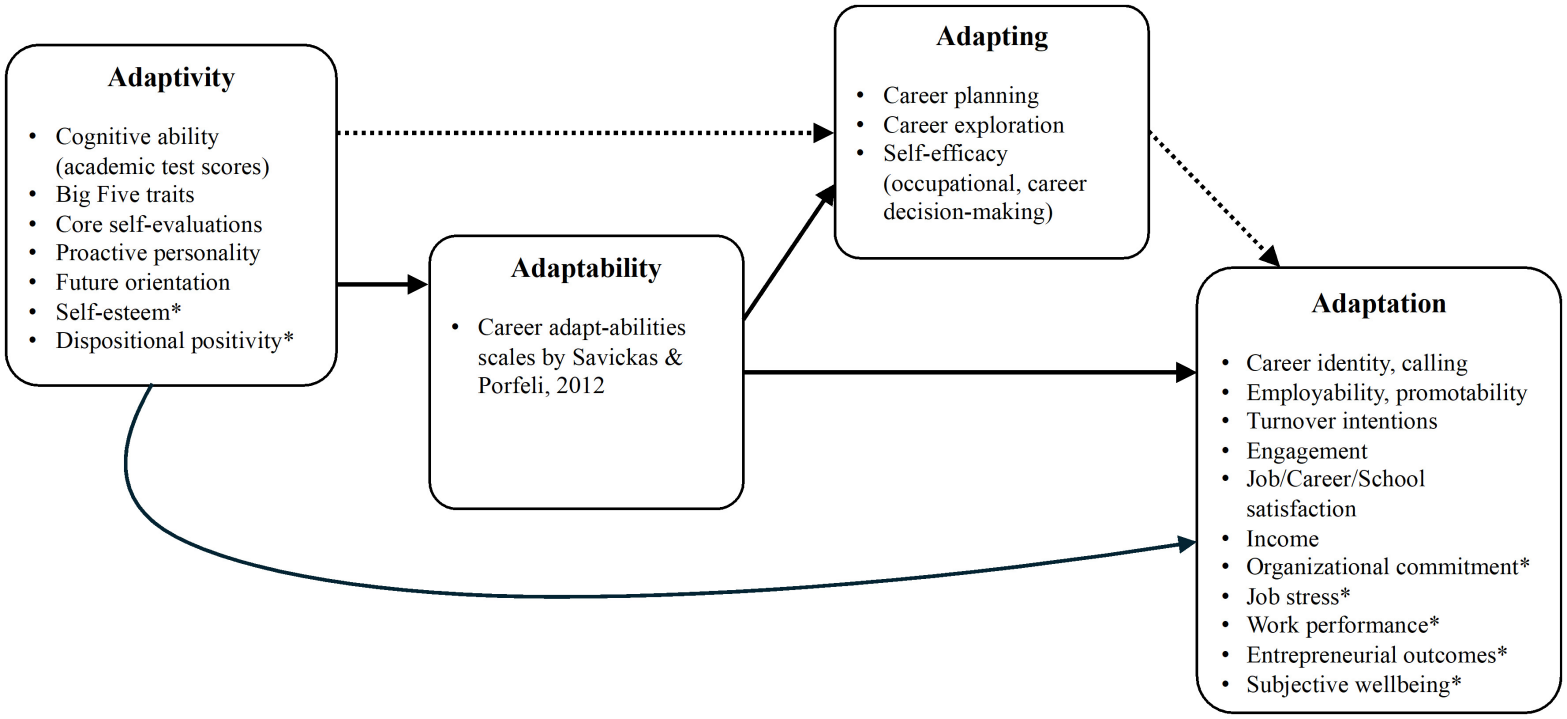
Adapting the career construction model of adaptation: examining relationships among the “four As”. This schematic diagram, adapted from the work of Rudolph et al. (2017), illustrates the application of Savickas and Porfeli's CCMA framework to examine relationships among the “four As” variables. Solid arrows represent examined associations, while dashed arrows represent unexamined associations. Asterisk (*) denotes measures that were not considered within the scope of this paper.

Despite its strengths, CCMA's comprehensiveness poses challenges in achieving a cohesive and consistent understanding of the adaptation process. A key limitation of extant research lies in the methodological variability in how the “four As” are operationalized. While adaptability is commonly measured using the career adapt-abilities scale (CAAS) developed by Savickas and Porfeli (2012), the other dimensions rely on more heterogeneous measures. For example, Rudolph et al.'s (2017) meta-analysis (see Figure 1) revealed that adaptivity has been assessed using diverse indicators such as cognitive ability, Big Five personality traits, core self-evaluations, proactive personality, and future orientations. Prior research has relied on individual measures of the “four As”, overlooking the shared variance across multiple instruments within each dimension. Theoretical discrepancies across measurement tools may capture only fragments of the “four As” rather than their full theoretical scope (Perera and McIlveen, 2017). This omission in the existing literature creates gaps in representation and limits the theoretical utility and conceptual clarity of the CCMA framework.

In addition, although the CCMA explicitly proposes a serial mediation among the “four As,” many existing studies—including Rudolph et al. (2017)—have not tested all possible interrelationships. Some studies (e.g., Rudolph et al., 2017; Zhou et al., 2016) employed multiple regression techniques to examine direct and indirect effects, which are suboptimal for capturing the full serial mediation structure embedded in the CCMA. Additionally, reported mediation models (e.g., Leung et al., 2022; Merino-Tejedor et al., 2016; Šverko and Babarović, 2019) have not consistently demonstrated the serial mediation pathway posited by the CCMA. In some cases, variables assumed to be conceptually linked show no significant association, raising concerns that such discrepancies in earlier studies stem more from fragmented measurement practices than from theoretical shortcomings.

### 1.2 Overview of studies

To address these challenges, the present research begins by identifying common factors underlying a range of assessments that capture constructs associated with each dimension of the Career Construction Model of Adaptation (CCMA). By modeling the common variance across validated measures, we move beyond fragmented operationalizations to offer a more conceptually integrated and empirically grounded representation of CCMA. Specifically, we build on Rudolph et al.'s (2017) meta-analytic framework—the most comprehensive synthesis to date, encompassing ninety studies and a wide range of validated measures. While recent meta-analyses (e.g., Stead et al., 2022; Vashisht et al., 2023; Wang and Dong, 2024; Wang et al., 2023; Yu et al., 2023) have provided valuable insights into specific facets of CCMA, they fall short of offering a unified theoretical account of its dimensional structure. Guided by Rudolph et al.'s findings, we identify and integrate widely used self-report measures to clarify the latent structure of each dimension, thereby enhancing both the theoretical precision and practical utility of the CCMA framework.

We selected validated measures for each dimension of the CCMA model and applied psychometric techniques to examine the shared variance among 17 scales representing the “four As.” Scale selection was guided by Rudolph et al.'s (2017) meta-analysis, with measures assigned to CCMA dimensions based on their theoretical alignment with the framework and their established use in the career development literature. Our objective was to assess the extent to which the resulting factors align with the theoretical structure proposed by Savickas and Porfeli (2012), thereby validating the model's dimensional integrity and offering deeper insight into career construction processes. We then analyzed the relationships among the extracted factors to test whether they replicate the serial mediation structure outlined in the CCMA framework.

We analyzed two distinct samples: working adults (Study 1) and university students (Study 2). Notably, many existing CCMA studies (e.g., Leung et al., 2022; Merino-Tejedor et al., 2016; Perera and McIlveen, 2017; Savickas et al., 2018; Šverko and Babarović, 2019; Zhou et al., 2016) that examined the full CCMA model have predominantly examined student samples, including high school and university students. Drawing on Super's (1973) career development theory, we recognize that students typically engage in tasks such as crystallizing and specifying career choices, while adults focus on stabilizing their careers or managing transitions (Savickas, 1994). These differences shape individuals' engagement with the underlying constructs of CCMA. For instance, Gianakos (1996) found that adults demonstrate higher career decision-making self-efficacy and are more likely to pursue further training or career changes to align with personal values, emphasizing autonomy and meaningful work. In contrast, students often prioritize extrinsic factors such as salary, job security, and promotion prospects, which shape their career-related decisions and strategies. By incorporating both students and working professionals, our study provides a more comprehensive understanding of how the CCMA operates across populations at distinct career development stages, each characterized by divergent motivations, competencies, and behaviors that warrant separate analyses.

## 2 Study 1

### 2.1 Methods

#### 2.1.1 Participants and procedures

We collected survey responses from 1,013 working adults in Singapore using an online survey administered via Qualtrics. A preliminary screening determined eligibility: participants had to (1) be Singaporean nationals aged 25 years−40 years, (2) make no more than one error on an instructed response item (Gummer et al., 2021; Kam and Chan, 2018; Muszyński, 2023; Shamon and Berning, 2020), and (3) complete at least 90% of the survey. Those who did not meet these criteria were redirected to the end of the survey with a brief explanation. We targeted the 25–40 age range because individuals in this developmental stage are typically in the establishment phase of their careers, a period characterized by the consolidation of professional identity and key career decision-making (Chen et al., 2020). This phase is particularly relevant for studying adaptability, as individuals often face transitions such as promotions, job changes, or shifts driven by personal or economic circumstances. To protect participant anonymity while allowing for potential follow-up, we assigned each respondent a unique anonymized identifier linked to their survey data, without collecting any personally identifiable information.

After following the above procedure, we retained 1,000 valid cases for the final analysis, 48.9% of whom were female, with a mean age of 32.76 years. In terms of education, 0.5% held less than a pre-university qualification, 86% held bachelor's degrees, 2.8% professional degrees, 10.5% master's degrees, and 0.2% doctorates. Participants identified ethnically as Chinese (80%), Indian (9.8%), Malay (7.9%), other Asian (2%), and Eurasian (0.2%). Most participants (95.5%) were employed full-time, while 0.9% worked part-time, 2.4% were self-employed, and 1.2% were unemployed or between jobs. Approximately 51.7% reported working in a single industry, whereas 48.3% reported employment across multiple sectors. Among employed respondents, 67.5% worked in the services sector, 18.4% in manufacturing, 12.3% in construction, and 1.8% in other industries. This distribution closely reflects Singapore's national workforce composition where the majority are employed in services-related occupations (Ministry of Manpower Singapore, 2025). Detailed demographic characteristics are presented in Table 1. On average, participants had 6.2 years of work experience (SD = 0.91).

**Table 1:** Study 1: demographic characteristics (*N* = 1,000).

Demographics	Category	*n*	%
Gender	Female	489	48.9
	Male	510	51.0
Education	Less than pre-university	5	0.5
	Bachelor's degree	860	86.0
	Professional degree	28	28.0
	Master's degree	105	10.5
	Doctorate	2	0.2
Ethnicity	Chinese	800	80.0
	Indian	98	9.8
	Malay	79	7.9
	Other Asian	20	2.0
	Eurasian	2	0.2
Employment status	Full-time	955	95.5
	Part-time	9	0.9
	Self-employed	24	24.0
	Unemployed/between jobs	12	1.2
Industry	Services	675	67.5
	Manufacturing	184	18.4
	Construction	123	12.3
	Other	18	1.8
Industry exposure	Single industry	517	51.7
	Multiple industry	483	48.3

Mean age = 32.76 years (SD = 4.33). Mean work experience = 6.20 years (SD = 0.91).

#### 2.1.2 Measures

We measured adaptivity (A1), adaptability (A2), adapting (A3), and adaptation (A4)—collectively referred to as the “four As”—using well-established measures frequently applied in CCMA-related research. We selected these measures based on theoretical grounding, empirical support, and relevance to the study context. Guided by insights from Rudolph et al.'s (2017) meta-analysis, we proposed a comprehensive set of variables to represent each dimension and facilitate factor extraction. All variables modeling the “four As” rely on self-reported data. We used largely the same instruments across Studies 1 and 2, with several sample-specific differences in measure selection. Specifically, four work-related measures (Protean and Boundaryless Career Attitude Scales, Work Self-Efficacy Scale, and Turnover Intention Scale) were administered only to working adults, whereas the Academic Satisfaction Scale was administered exclusively to university students. These distinctions are indicated in Table 2.

**Table 2:** List of measures employed in studies 1 and 2.

Dimension	Measure
Adaptivity (A1)	Cognitive flexibility scale (CFS; Martin and Rubin, 1995)
	The big five inventory 10 item scale (BFI-10; Rammstedt and John, 2007)
	Proactive personality scale (PPS; Seibert et al., 1999)
	Core self-evaluations scale (CSES; Judge et al., 2003)
	Future work self salience (FWS; Strauss et al., 2012)
Adaptability (A2)	Career adapt-abilities scale – international version 2.0 (CAAS; Savickas and Porfeli, 2012)
	^*^Protean career attitude scale (PCAS; Briscoe et al., 2006)
	^*^Boundaryless career attitude scale (BCAS; Briscoe et al., 2006)
Adapting (A3)	Proactive career behavior scale (PCBS; Strauss et al., 2012)
	“Getting ready for your next job” Inventory (JSE; Wanberg et al., 2010)
	Career decision ambiguity tolerance scale (CDATS; Xu and Tracey, 2015)
Adaptation (A4)	Career decidedness scale (CADS; Lounsbury et al., 2005)
	“My vocational situation” (MVS; Holland et al., 1980)
	Self-perceived employability scale (SPE; Rothwell and Arnold, 2007)
	^*^Work self-efficacy scale (WSES; Avallone et al., 2007)
	^*^Turnover intention scale (TIS; Bothma and Roodt, 2013)
	^†^Academic satisfaction scale (AS; Lent et al., 2005)

The names of the measures used in Studies 1 and 2 are presented here; detailed information, including sample items and scoring instructions, can be found in the Supplementary material.

^*^Scales included only in Study 1.

^†^Scales included only in Study 2.

#### 2.1.3 Analysis

We used the same statistical procedures for both Studies 1 and 2. We performed confirmatory factor analysis (CFA) with maximum likelihood estimation to establish the construct validity of the latent factors for all measures. Factor loadings, path coefficients, variance, and measures of model fit were estimated using R version 4.2.1 (R Core Team, R Foundation for Statistical Computing, Vienna). The model's goodness of fit was assessed using several measures, including absolute fit indices, i.e., the chi-square test and the standardized root mean squared residual (SRMR), and relative fit indices, i.e., the comparative fit index (CFI), Akaike information criterion (AIC), and sample-size adjusted Bayesian information criterion (SABIC). We adhered to well-established guidelines (CFI > 0.90, Bentler, 1990; SRMR ≤ 0.08, Hu and Bentler, 1999) while using these fit indices. For model comparison, we prioritized models with lower AIC and SABIC values.

Savickas and Porfeli's (2012) CCMA posits that *adaptivity* positively influences *adaptability*, which in turn positively affects *adapting* responses and *adaptation* outcomes. Building on CCMA, we employed a serial mediation model to examine the intricate relationships among the “four As” and deepen our understanding of their effects on individuals' career development and transitions.

To evaluate the model, we compared it against alternative models that examined potential scenarios where: (a) *adaptability* fully or partially mediates the relationship between *adaptivity* and *adapting* responses; (b) *adaptability* fully or partially mediates the relationship between *adaptivity* and *adaptation*; (c) *adapting* responses fully or partially mediate the relationship between *adaptivity* and *adaptation*; and (d) *adapting* responses fully or partially mediate the relationship between *adaptability* and *adaptation*. We employed a biased-corrected bootstrapping technique (Preacher et al., 2007; Shrout and Bolger, 2002) with 3,000 bootstrap samples to test the models by computing the indirect effects and their 95% confidence intervals.

### 2.2 Results

#### 2.2.1 Normality of data

We examined the distributions of all items included in the data analysis, following Kim's (2013) criteria for evaluating normality in large samples (*n* > 300). We calculated the absolute values of skewness and kurtosis for all variables. Skewness values fell within the range of −2–+2, and kurtosis values ranged from −7 to +7, suggesting no significant violation of the normality assumption in our data.

#### 2.2.2 Common method bias assessment and factor structure analysis

We used CFA to assess the construct validity of the included measures. By adhering to the proposed factor structure, we aimed to validate the measures, thereby enhancing the robustness of our findings while also accounting for the demographic nuances of our study context. All final factor models achieved acceptable fit indices, with CFI values ≥0.90 (Bentler, 1990) and SRMR values ≤ .08 (Hu and Bentler, 1999). Table 3 provides a detailed summary of all CFA results, including the number of items and factors removed for each measure.

**Table 3:** Confirmatory factor analysis (CFA) results for the measures.

Model	Number of items	χ^2^	df	χ^2^/df	CFI	SRMR	AIC	SABIC	Remark
CFS two-factor	9	257.12	26	9.89	0.92	0.06	20,454.20	20,487.10	remove items 2, 8, 12
BFI-10 three-factor	6	62.44	9	7.52	0.97	0.03	15,646.53	15,667.31	remove agreeableness and conscientiousness factors
PPS one-factor	10	378.27	36	10.51	0.95	0.04	23,174.64	23,207.54	
CSES one-factor	12	563.12	54	10.43	0.91	0.05	22,703.72	22,745.28	
FWS one-factor	5	374.91	5	74.98	0.90	0.05	9,102.18	9,151.26	
CAAS four first-order factor, one second-order factor	24	1,632.95	248	6.58	0.90	0.05	52,489.25	52,579.30	
PCAS two-factor	14	845.33	76	11.12	0.90	0.06	29,272.26	29,322.48	
BCAS two-factor	12	641.70	53	12.11	0.92	0.06	27,154.35	27,218.42	remove item 8
PCBS four-factor	13	356.02	59	6.03	0.96	0.05	24,396.60	24,452.02	
JSE one-factor	8	259.28	20	12.96	0.92	0.05	17,415.77	17,443.48	remove items 2, 4, 5
CDATS three-factor	12	633.01	51	12.41	0.90	0.06	32,297.09	32,343.84	remove items 5, 6, 10, 12, 17, 18
CADS one-factor	6	118.64	9	13.18	0.96	0.04	11,967.46	11,988.24	
MVS one-factor	17	1,064.84	119	8.95	0.91	0.05	37,336.97	37,395.85	remove item 18
SPE two-factor	11	493.37	43	11.47	0.91	0.06	21,035.83	21,075.66	
WSES one-factor	9	343.49	27	12.72	0.90	0.05	19,078.45	19,125.20	remove item 10
TIS one-factor	6	220.79	9	23.99	0.93	0.06	14,385.40	14,416.57	
AS one-factor^†^	7	144.07	14	10.29	0.90	0.06	4,958.74	4,966.78	

CFA results are based on Study 1 (*N* = 1,000), except for the Academic Satisfaction Scale (^†^), which was estimated using Study 2 (*N* = 323). For scales where items were removed, item numbers refer to the original numbering in the published scale. CFI, Comparative fit index; SRMR, standardized root mean squared residual; AIC, akaike information criterion; SABIC, sample-size adjusted Bayesian Information criterion.

We successfully replicated the factor model for most measures, although some scales required adjustments to achieve optimal model fit. Specifically, for scales such as cognitive flexibility, boundaryless career attitude, career decision ambiguity tolerance, job search efficacy, vocational identity, and work self-efficacy, we removed items that exhibited comparatively lower factor loadings within their respective scales. Rather than applying a fixed cutoff (e.g., loading < 0.30; Shevlin and Miles, 1998), item removal was guided by overall model fit indices and the goal of enhancing the robustness and interpretability of the factor structure. In certain cases, adjustments went beyond item removal and involved restructuring factors. For instance, the conscientiousness and agreeableness factors of the BFI-10 were excluded due to their limited applicability to our dataset. Although the BFI-10 measures well-established constructs, reliability concerns emerged in our sample, with low Cronbach's alpha coefficients observed for agreeableness (α = 0.27) and conscientiousness (α = 0.04). Prior research has similar identified issues with the agreeableness dimension, including weak or negative correlations (Lu and Cui, 2022; Ludeke and Larsen, 2017) and cross-loadings across factors (Levinsky et al., 2019; Steyn and Ndofirepi, 2022). Notably, Levinsky et al. (2019) combined agreeableness and conscientiousness due to poor reliability. These findings highlight the need for further refinement of the scale. Additionally, we merged multiple factors into a single factor where appropriate. For example, while the original work self-efficacy scale (Pepe et al., 2010) proposed a two-factor structure, our data supported a simpler, unified one-factor structure that demonstrated satisfactory model fit. These refinements ensured that the final models better aligned with the characteristics of our sample.

We assessed the potential impact of common method bias (CMB) using the unmeasured latent variable technique. A latent common method factor was added to the measurement model, loaded on all observed indicators, and constrained to be orthogonal to the substantive constructs (Williams et al., 1989). The method factor explained 35.7% of the total variance shared by the variables (χ^2^ = 37,383.88, df = 14,351; CFI = 0.82; SRMR = 0.06), while standardized loadings on the method factor ranged from 0.00 to 0.63, most of which were below 0.40, suggesting that CMB does not appear to fully account for the observed relationships. In addition to the statistical test, we implemented several procedural remedies (Podsakoff et al., 2003, 2024) during the study design stage to minimize potential CMB. These included: (1) varying Likert-type response formats (e.g., five-point “extent” vs. seven-point “agreement” scales), (2) incorporating reverse-coded items, and (3) ensuring participant anonymity and excluding any personally identifiable information, thereby reducing evaluation apprehension and social desirability concerns.

#### 2.2.3 Descriptive statistics

**Figure 2:**
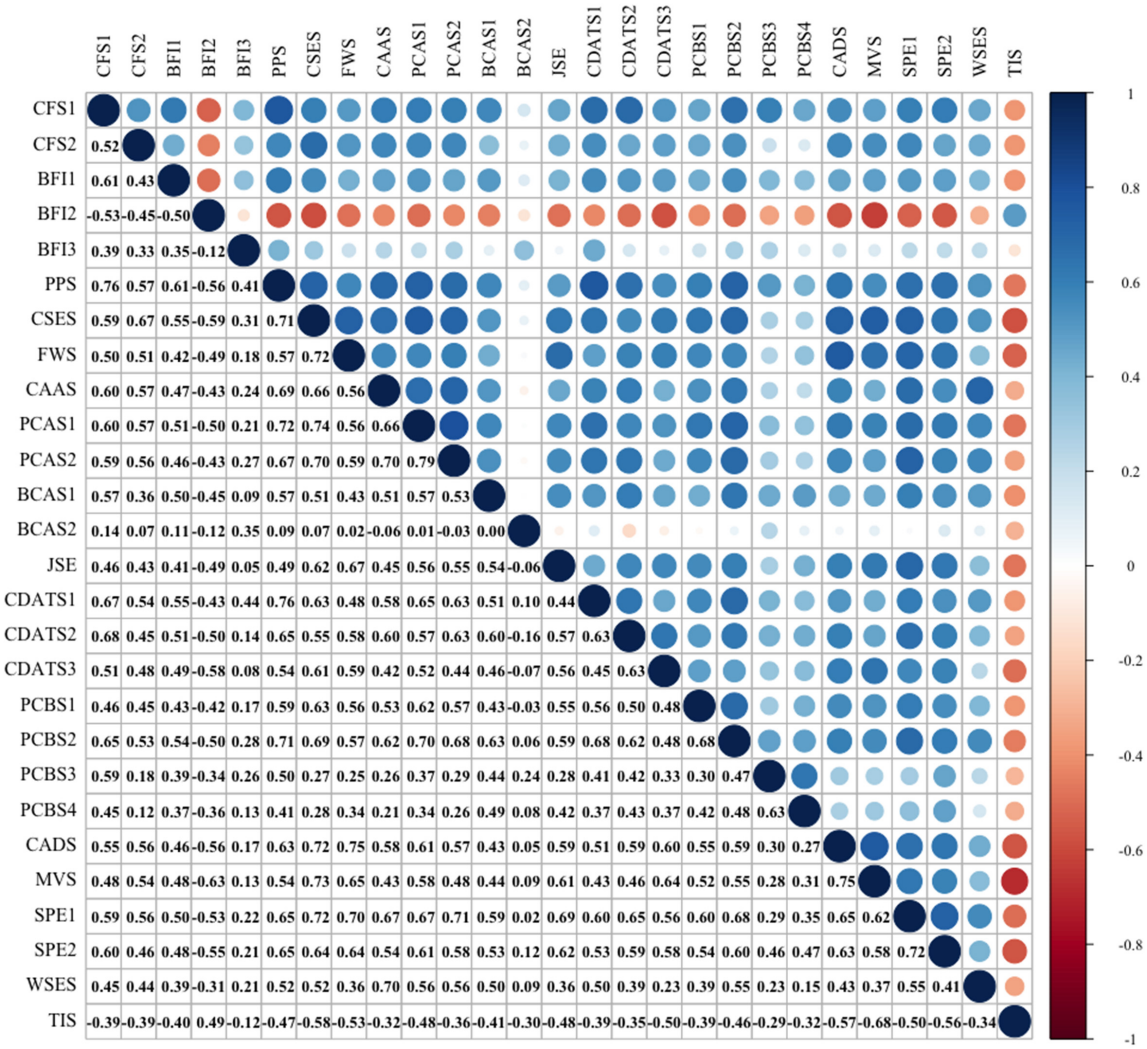
Correlations: Study 1. *N*, 1,000 working adults in Singapore; CFS1, cognitive flexibility: willingness to be flexible; CFS2, cognitive flexibility: efficacy in being flexible; BFI1, big five: extraversion; BFI2, big five: neuroticism; BFI3, big five: openness; PPS, proactive personality; CSES, core self-evaluations; FWS, future work self salience; CAAS, career adapt-abilities; PCAS1, protean career attitude: self-directed; PCAS2, protean career attitude: value-directed; BCAS1, boundaryless career attitude: boundaryless mindset; BCAS2, boundaryless career attitude: mobility preference; JSE, job search efficacy; CDATS1, career decision ambiguity tolerance: preference; CDATS2, career decision ambiguity tolerance: tolerance; CDATS3, career decision ambiguity tolerance: aversion; PCBS1, proactive career behavior: career planning; PCBS2, proactive career behavior: skill development; PCBS3, proactive career behavior: career consultation; PCBS4, proactive career behavior: network building; CADS, career decidedness; MVS, my vocational situation” (MVS); SPE1, self-perceived employability: external employability; SPE2, self-perceived employability: internal employability; WSES, work self-efficacy; TIS, turnover intention.

Table 4 presents the descriptive statistics (means, standard deviations) and reliability coefficients, while Figure 2 shows the correlations among the variables. All measures demonstrated acceptable internal consistency reliability, with Cronbach's alpha coefficients exceeding 0.60, (Nunnally and Bernstein, 1994).

**Table 4:** Study 1: descriptive statistics and reliabilities (*N* = 1,000).

No.	Scale	Number of items	*M*	SD	α
1	CFS: willingness to be flexible	6	4.76	0.61	0.82
2	CFS: efficacy in being flexible	3	4.46	0.80	0.72
3	BFI: extraversion	2	3.71	0.96	0.79
4	BFI: neuroticism	2	2.22	0.84	0.73
5	BFI: openness	2	3.74	0.95	0.69
6	PPS: proactive personality	10	5.54	0.81	0.93
7	CSES: core self-evaluation	12	3.90	0.56	0.91
8	FWS: future work self	5	4.04	0.71	0.91
9	CAAS: career adaptability	24	3.70	0.65	0.95
10	PCAS: self-directed attitude	8	4.00	0.62	0.88
11	PCAS: value-directed attitude	6	3.74	0.71	0.86
12	BCAS: boundaryless mindset	7	3.52	0.79	0.91
13	BCAS: mobility preferences	5	2.89	0.86	0.87
14	JSE: job search efficacy	8	3.83	0.60	0.85
15	CDATS: preference	4	5.60	0.79	0.87
16	CDATS: tolerance	4	5.15	0.91	0.79
17	CDATS: aversion	4	4.45	1.11	0.76
18	PCBS: career planning	4	4.16	0.62	0.82
19	PCBS: skill development	3	4.10	0.60	0.81
20	PCBS: career consultation	3	3.33	1.02	0.93
21	PCBS: network building	3	3.89	0.72	0.84
22	CADS: career decidedness	6	3.93	0.67	0.88
23	MVS: vocational identity	17	3.98	0.69	0.94
24	SPE: external employability	7	4.02	0.57	0.88
25	SPE: internal employability	4	3.82	0.61	0.72
26	WSES: work self-efficacy	9	3.95	0.56	0.86
27	TIS: turnover intention	6	2.33	0.75	0.82

Correlation analyses revealed that most variables in this study exhibited statistically significant associations. However, mobility preference (a sub factor of boundaryless career attitude) did not form meaningful correlations with other variables, including future work self, career adaptability, protean career attitudes, boundaryless mindset, proactive career behaviors, job search efficacy, career decidedness, and perceived external employability. Similarly, Big Five openness to experience showed non-significant correlations with job search efficacy. Regarding directionality, most variables were positively correlated with one another. As expected, neuroticism and turnover intention consistently demonstrated negative correlations with the other variables in the study.

#### 2.2.4 Extracting the “four As”

Building on Rudolph et al.'s (2017) meta-analysis, we computed standardized composite scores for each measure based on the CFA results and categorized these composites into four distinct groups corresponding to the *adaptive, adaptability, adapting*, and *adaptation* dimensions. We then performed a separate one-factor CFA for each group.

**Figure 3:**
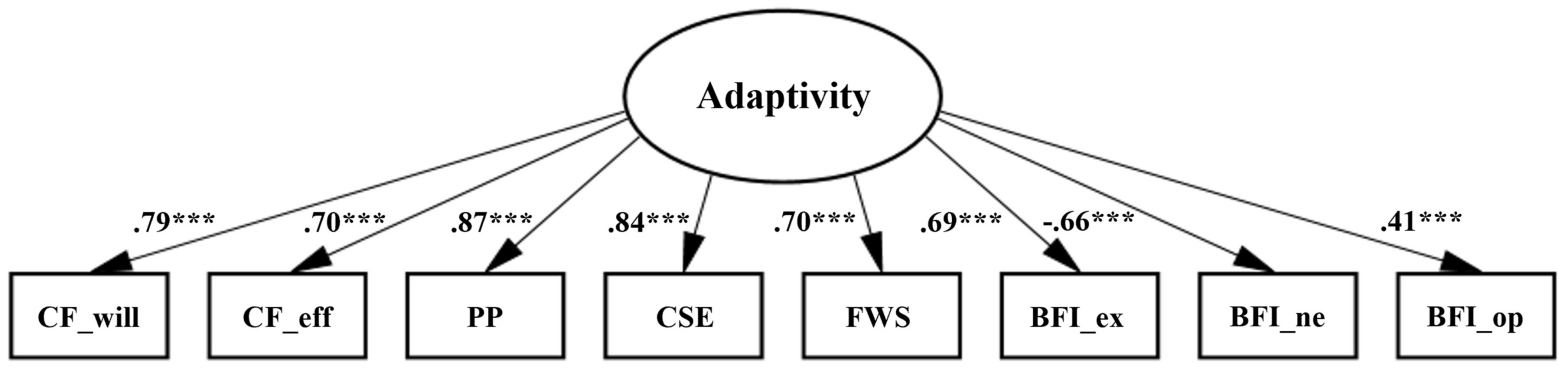
One-factor parceling model of adaptivity (A1): study 1. *N* = 1,000. Standardized factor loadings are reported in this figure. *** *p* ≤ 0.001. CF_will, cognitive flexibility: willingness to be flexible; CF_eff, cognitive flexibility: efficacy in being flexible; PP, proactive personality; CSE, core self-evaluations; FWS, future work self salience; BFI_ex, big five: extraversion; BFI_ne, big five: neuroticism; BFI_op, big five: openness.

To examine the factor structure of A1, composite scores from measures, including cognitive flexibility, Big Five, proactive personality, core self-evaluations, and future work self (total number of composites = 8; see Figure 3) were used in a one-factor model. The results indicated adequate model fit (χ^2^ = 472.22, df = 20; CFI = 0.90; SRMR = 0.05), based on conventional fit criteria (e.g., Bentler, 1990; Hu and Bentler, 1999).

**Figure 4:**
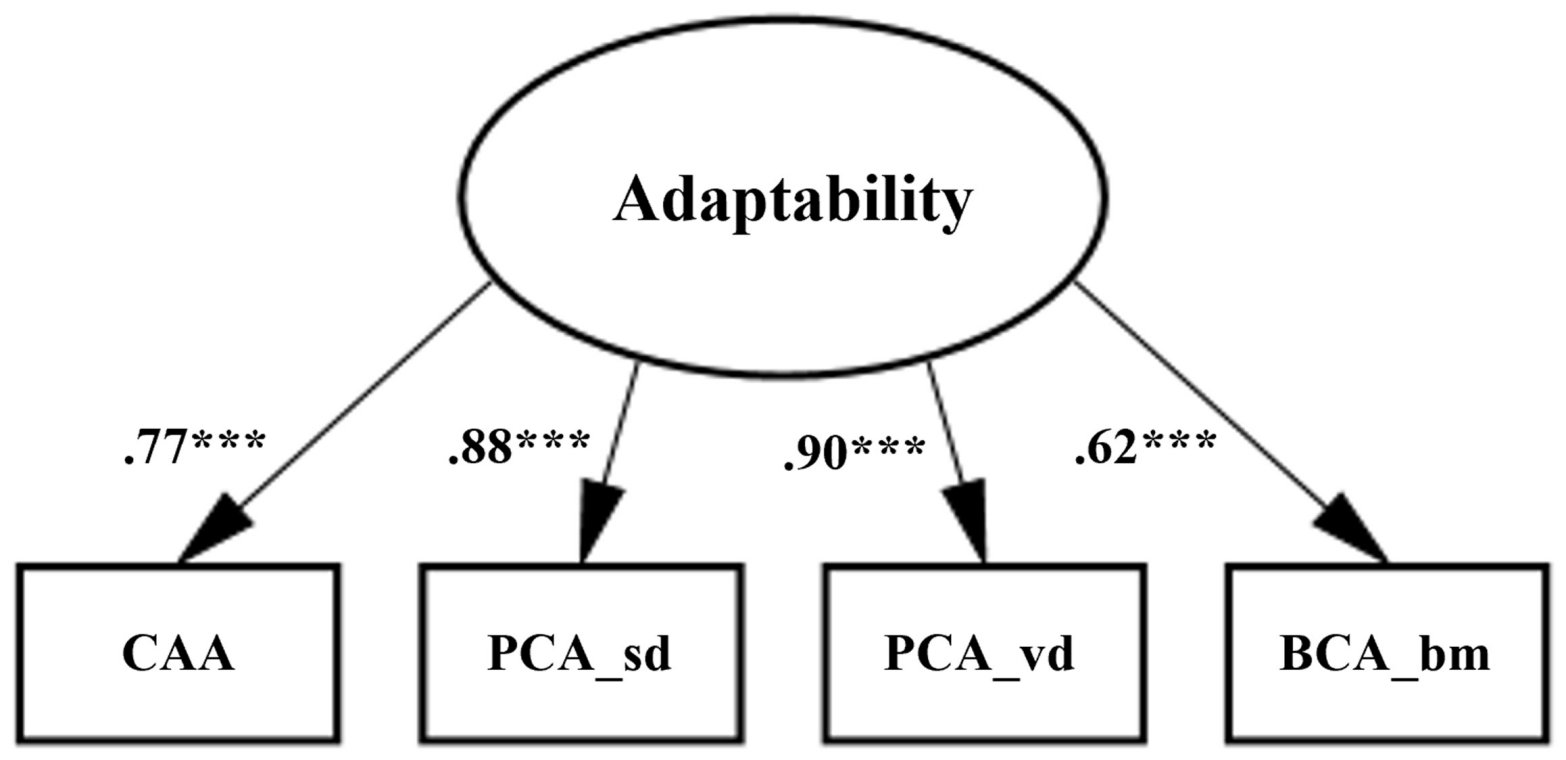
One-factor parceling model of adaptability (A2): study 1. *N* = 1,000. Standardized factor loadings are reported in this figure. *** *p* ≤ 0.001. CAA, career adapt-abilities; PCA_sd, protean career attitude: self-directed; PCAS_vd, protean career attitude: value-directed; BCA_bm, boundaryless career attitude: boundaryless mindset.

In exploring the dimension of A2, the original CCMA framework was adopted, using Savickas and Porfeli's (2012) career adapt-abilities inventory (χ^2^ = 1,632.95, df = 248; CFI = 0.90; SRMR = 0.05) as the sole representative measure for this second dimension. On the other hand, to broaden the adaptability dimension beyond the scope of the career adaptability measure, we incorporated protean and boundaryless career attitudes, both of which also emphasize the ability to adapt flexibly to work-related contingencies (Hall, 2002; Wiernik and Kostal, 2019). The goal was to examine whether the synergy of these constructs (total number of composites = 4; see Figure 4) could augment the predictive value of the extracted A2. The CFA results for the combination of the three measures were χ^2^ = 20.60, df = 2, CFI = 0.99, and SRMR = 0.02. It is important to note that organizational mobility preference was excluded from the model based on correlation analysis, which indicated non-significant associations with career adaptability, protean attitudes, and boundaryless mindset factors. This aligns with prior research (e.g., Briscoe et al., 2006; Çakmak-Otluoglu, 2012; Wiernik and Kostal, 2019) which underscores mobility preference as a distinct construct, separate from other protean and boundaryless career attitudes.

**Figure 5:**
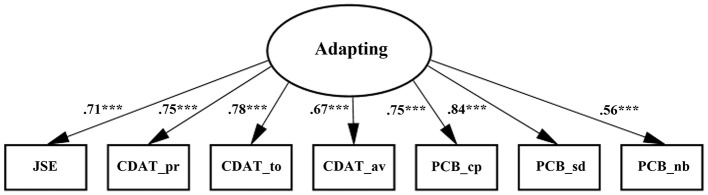
One-factor parceling model of adapting (A3): study 1. *N* = 1,000. Standardized factor loadings are reported in this figure. *** *p* ≤ 0.001. JSE, job search efficacy; CDAT_pr, career decision ambiguity tolerance: preference; CDAT_to, career decision ambiguity tolerance: tolerance; CDAT_av, career decision ambiguity tolerance: aversion; PCB_cp, proactive career behavior: career planning; PCB_sd, proactive career behavior: skill development; PCB_nb, proactive career behavior: network building.

To extract the third factor (A3), all composite scale scores for proactive career behaviors, job search efficacy, and career decision ambiguity (total number of composites = 8) were initially included in the CFA, which yielded a poor-fitting model (χ^2^ = 620.31, df = 20; CFI = 0.85; SRMR = 0.07), based on conventional criteria (e.g., CFI > 0.90, Bentler, 1990). Follow-up examination revealed that career consultation (a component of proactive career behaviors) exhibited a lower factor loading compared to other composites. This finding suggests that the career consultation component may contribute less to the A3, potentially impacting the overall model fit. Therefore, the consultation composite score was excluded from the model (see Figure 5), and the subsequent analysis demonstrated a significant improvement in the model fit (total number of composites = 7; χ^2^ = 289.18, df = 14; CFI = 0.92; SRMR = 0.05).

**Figure 6:**
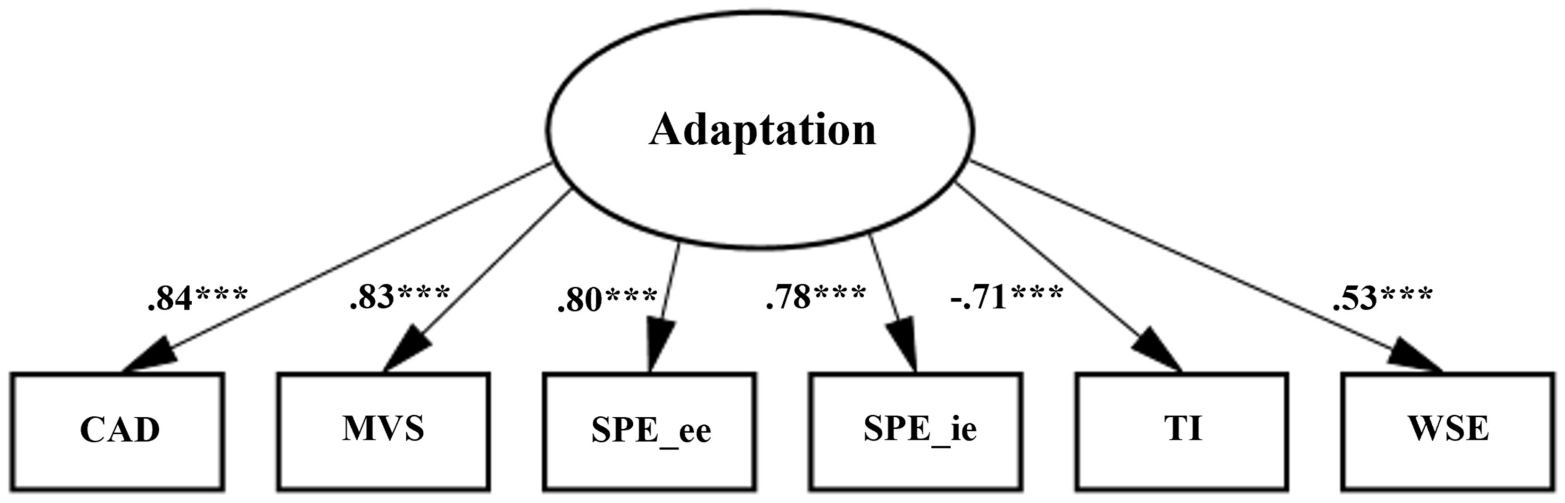
One-factor parceling model of adaptation (A4): study 1. *N* = 1,000. Standardized factor loadings are reported in this figure. *** *p* ≤ 0.001. CAD, career decidedness; MVS, my vocational situation; SPE_ee, self-perceived employability: external employability; SPE_ie, self perceived employability: internal employability; TI, turnover intention; WSE, work self-efficacy.

Finally, composite scores from career decidedness, vocational identity, perceived employability, work self efficacy, and turnover intention measures (total number of composites = 6; see Figure 6) were used to delineate the A4 factor. The results of the CFA indicated acceptable model fit (χ^2^ = 323.10, df = 9; CFI = 0.91; SRMR = 0.05).

Factor scores of the “four As” were subsequently computed from each factor model and used in the mediation analysis. The estimated factor scores of the “four As” exhibited significant associations with each other, with correlation coefficients ranging between 0.76 and 0.87 (see Table 5).

**Table 5:** Study 1: correlations among the “four As”.

No.	Factor	1	2	3	4
1	Adaptivity	—			
2	Adaptability	0.83^***^	—		
3	Adapting	0.87^***^	0.82^***^	—	
4	Adaptation	0.85^***^	0.76^***^	0.82^***^	—

*N* = 1,000.

^***^ *p* ≤ 0.001.

#### 2.2.5 Serial mediation model of the relationships among the “four As”

One of the study goals was to determine whether the “four As” extracted from various measures could replicate the serial mediation model portrayed in CCMA. For this purpose, we first tested a partial mediation model in which A1 predicted A2, which in turn predicted A3 and subsequently A4 (A1 → A2 → A3 → A4). Additionally, we examined several alternative models to further evaluate and establish the robustness of the main model. The alternative models included correlational models with only bidirectional arrows among the “four As” and full mediation models that assumed direct effects between factors. Detailed fit statistics for both main and alternative models are presented in Table 6. Notably, the path models with partial mediation were just identified (saturated), yielding uninformative fit indices (χ^2^ = 0, df = 0, CFI = 1, SRMR = 0). Because these fit indices lack meaningful interpretive value, we relied on AIC and SABIC for relative model comparison.

**Table 6:** Study 1: fit indices of the serial mediation models.

Model	χ^2^	*df*	χ^2^/*df*	CFI	SRMR	AIC	SABIC
With CAA as the sole representative of the adaptability dimension							
Correlational path model	0.00	0	N/A	1.00	0.00	7,464.22	7,481.53
Structural model: full mediation	1,131.78	3	377.26	0.68	0.19	5,816.49	5,826.88
Structural model: partial mediation	0.00	0	N/A	1.00	0.00	4,710.48	4,726.07
With CAA, PCA, and BM to represent the adaptability dimension							
Correlational path model	0.00	0	N/A	1.00	0.00	6,893.23	6,910.55
Structural model: full mediation	740.97	3	246.99	0.82	0.12	4,857.30	4,867.69
Structural model: partial mediation (final)	0.00	0	N/A	1.00	0.00	4,139.50	4,155.08

*N* = 1,000. CFI, comparative fit index; SRMR, standardized root mean squared residual; AIC, akaike information criterion; SABIC, sample-size adjusted Bayesian information criterion.

Our analysis to determine whether career adaptability alone sufficiently represents the *adaptability* dimension of the CCMA suggested that models with the A2 factor—including career adapt-abilities, protean career attitudes, and boundaryless mindset scales (AIC = 4,123.35, SABIC = 4,138.93)—outperformed those where only the CAAS scale represented *adaptability* (AIC = 4,697.41, SABIC = 4,713.00). These results suggest that incorporating protean career attitudes and boundaryless mindset may strengthen the structural representation of the *adaptability* factor. The proposed model also showed the best fit to the data. Specifically, models assuming (i) only correlations among the factors (AIC = 6,877.52, SABIC = 6,877.52) and (ii) full mediation (AIC = 4,858.31, SABIC = 4,868.70) showed higher AIC and SABIC, indicating poorer fit. Our findings align with evidence that protean career attitudes and boundaryless mindset are theoretically overlapped and empirically associated with career adaptability (e.g., Bernardo and Salanga, 2019; Hong et al., 2022; Inkson, 2006; Kundi et al., 2025; Rodrigues et al., 2019; Stauffer et al., 2019). These constructs represent a cluster of contemporary career orientations that equip individuals to navigate turbulent labor markets. Ultimately, these results are consistent with the serial mediation relationships among the *adaptivity, adaptability, adapting*, and *adaptation* dimensions as validated by Rudolph et al.'s (2017) meta-analysis.

**Figure 7:**
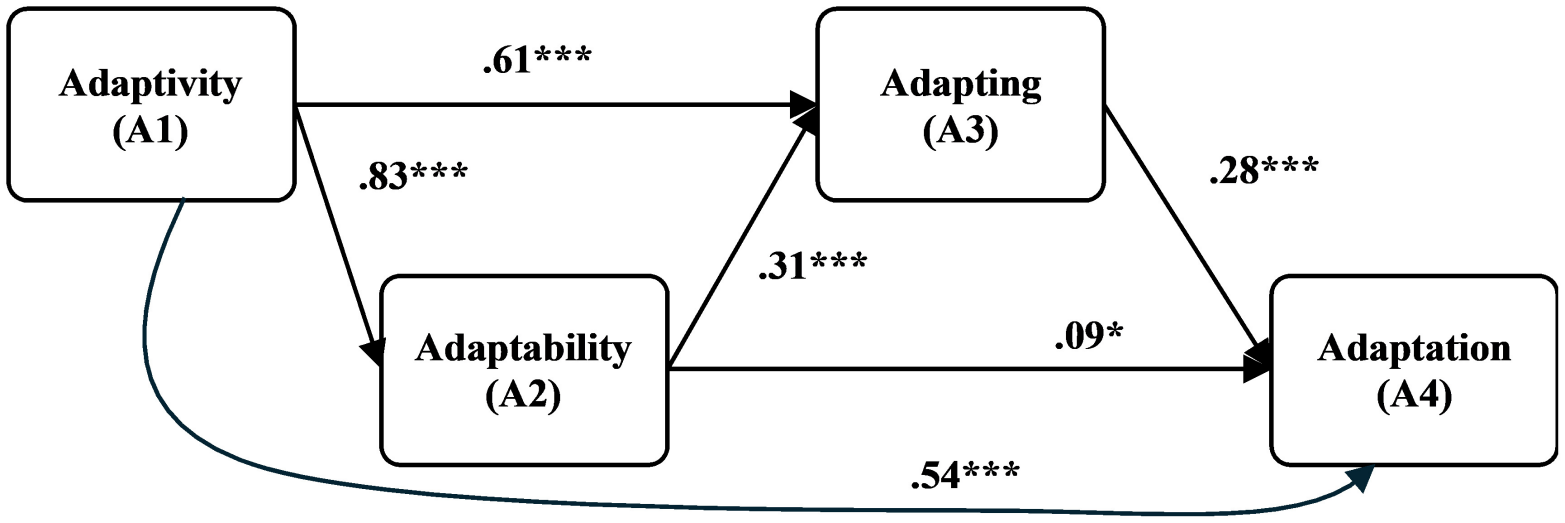
Serial mediation model of the “four As”: Study 1. *N* = 1,000. *** *p* ≤ 0.001; *p ≤ 0.05.

Serial mediation analysis of our final CCMA model (see Figure 7) revealed positive associations among all the “four As.” The results also indicate that the overall indirect effect of A1 on A4 via A2 and A3 factors was significant (β = 0.07, 95% CI = 0.05; 0.09). More specifically, the indirect effects of: (a) A1 on A3 through A2 (β = 0.26, 95% CI = 0.20; 0.31), (b) A1 on A4 through A2 (β = 0.07, 95% CI = 0.01; 0.13), (c) A1 on A4 through A3 (β = 0.16, 95% CI = 0.11; 0.22), and finally (d) A2 on A4 through A3 (β = 0.08, 95% CI = 0.06; 0.11) were all significant, as their 95% confidence intervals did not include zero (Preacher et al., 2007). Table 7 presents more details about the bootstrap results for the indirect effects of the tested mediation model.

**Table 7:** Study 1: bootstrap results for indirect effects.

		BCa 95% CI	
Indirect effects	β	Lower (95% BCa CI)	Upper (95% BCa CI)
A1 → A2 → A3 → A4	0.07^*^	0.05	0.10
A1 → A2 → A4	0.07^***^	0.01	0.13
A1 → A3 → A4	0.17^***^	0.11	0.22
A1 → A2 → A3	0.26^***^	0.20	0.31
A2 → A3 → A4	0.09^***^	0.06	0.12

*N* = 1,000. BCa, Bias-corrected and accelerated bootstrap interval. ^***^p ≤ 0.001; ^*^p ≤ 0.05.

## 3 Study 2

### 3.1 Methods

#### 3.1.1 Participants and procedures

We recruited 430 university students who were Singaporean nationals. To determine eligibility, we required participants to: (1) be enrolled in a local university, (2) commit no more than one error on an instructed response item (Gummer et al., 2021; Kam and Chan, 2018; Muszyński, 2023; Shamon and Berning, 2020), and (3) complete at least 80% of the survey. We set this lower completion threshold for Study 2 (compared to the 90% threshold used in Study 1) because Study 2 was administered under supervision. This adjustment allowed us to maintain data quality while preserving a sufficiently large sample for a comprehensive assessment of cognitive and career-related variables.

After excluding 74 participants who withdrew and 33 who failed to meet eligibility criteria, we retained 323 participants for analysis. Among them, 56.3% identified as female, with a mean age of 23.91 years (range = 20–31). Participants were distributed across academic years as follows: 13.9% in Year 1, 18.0% in Year 2, 40.6% in Year 3, and 27.5% in Year 4 or above. The majority identified as Chinese (89.8%), followed by Malay (4.3%) and Indian (4.0%). The remaining participants (1.9%) identified with other ethnicities. Participants were drawn from Science, Technology, Engineering, and Mathematics (STEM; 48.0%), non-STEM (44.9%), and mixed disciplines (5.0%). Detailed demographic characteristics are presented in Table 8.

**Table 8:** Study 2: demographic characteristics (*N* = 323).

Demographics	Category	*n*	%
Gender	Female	182	56.3
	Male	141	43.7
Ethnicity	Chinese	290	89.8
	Indian	13	4.0
	Malay	14	4.3
	Other ethnicities	6	1.9
Academic year	Year 1	45	13.9
	Year 2	58	18.0
	Year 3	131	40.6
	≥ Year 4	89	27.5
Academic discipline	STEM	155	48.0
	Non-STEM	145	44.9
	Mixed	16	5.0

Mean age = 23.91 years (SD = 1.67). STEM, science, technology, engineering, and mathematics.

As in Study 1, we administered the survey online via Qualtrics, allowing participants to complete it from home. We recruited participants through multiple channels, including the university subject pool, social media platforms, and campus posters placed in high-traffic locations. After registration, we sent participants an automated email with a link to the screening questionnaire and a unique identifier. We then contacted eligible individuals by email, guided them through the electronic consent process, and invited them to complete the main survey. After they submitted their responses, we provided a debriefing that outlined the study's purpose and thanked them for their participation.

#### 3.1.2 Measures

We used modified measures in Study 2 to better align with the specific context of this population. For a detailed description of the additional measures included in Study 2, please refer to Table 2.

### 3.2 Results

#### 3.2.1 Normality of data

We examined the distributions of all items following the same procedures as in Study 1. All items showed skewness values within the range of −2–+2 and kurtosis values between −7 and +7 (Kim, 2013), indicating that the data followed a normal distribution.

#### 3.2.2 Common method bias assessment

In Study 2 (*N* = 323), we computed composite scores for the variables based on the factor structure established in Study 1 (*N* = 1,000), leveraging the larger sample size in Study 1. Procedural remedies (Podsakoff et al., 2003, 2024) were implemented as described in Study 1. To evaluate the potential impact of CMB, we applied the same unmeasured latent variable technique as in Study 1. The method factor accounted for 16.3% of the total variance shared by the variables (χ^2^ = 15,788.93, df = 8,967; CFI = 0.73; SRMR = 0.06), while standardized loadings on the method factor ranged from 0.00 to 0.65, most of which were below 0.40, consistent with the pattern observed in Study 1.

#### 3.2.3 Descriptive statistics

**Figure 8:**
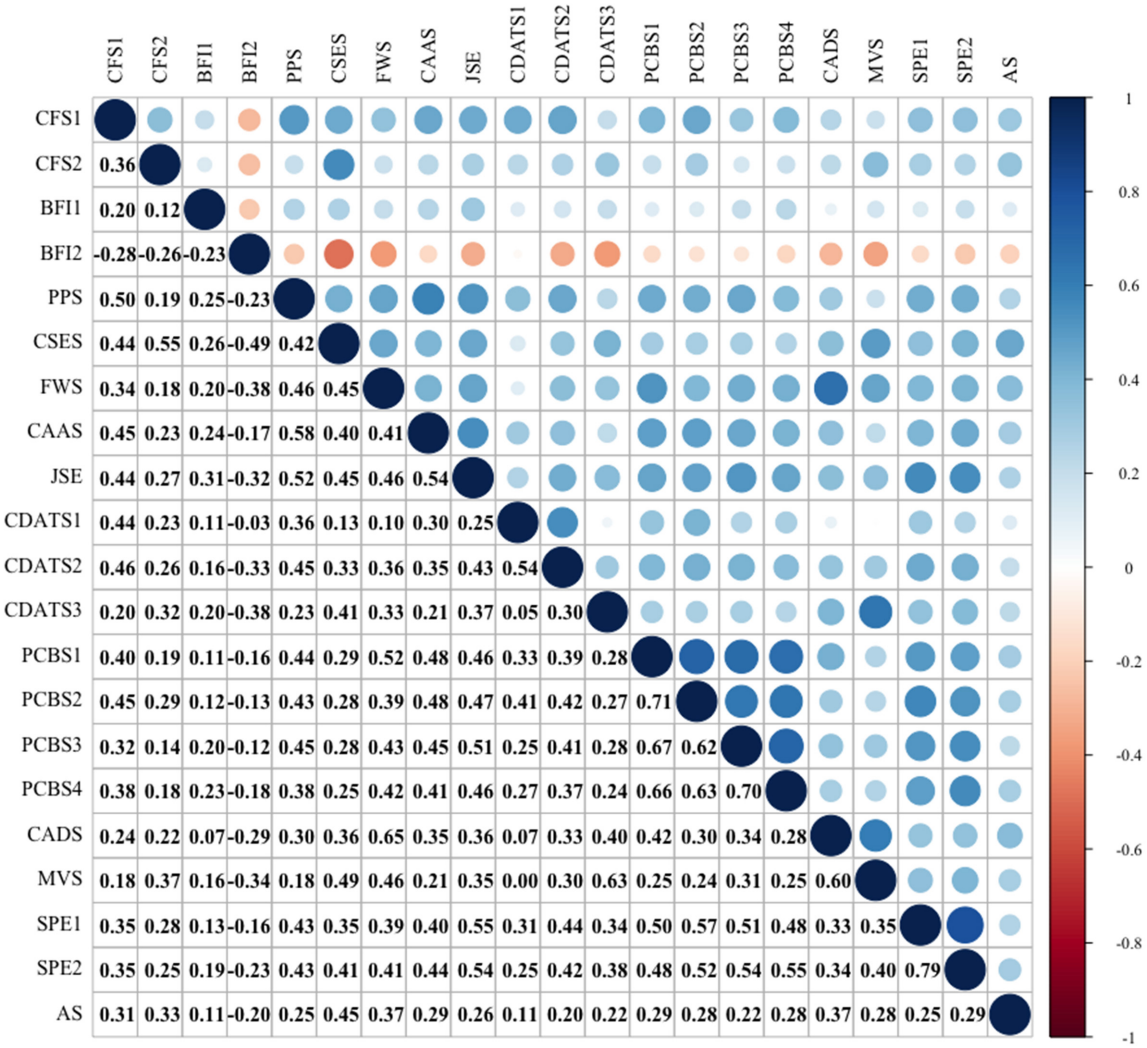
Correlations: study 2. *N* = 323 university student in Singapore. CFS1, cognitive flexibility: willingness to be flexible; CFS2, cognitive flexibility: efficacy in being flexible; BFI1, big five: extraversion; BFI2, big five: neuroticism; PPS, proactive personality; CSES, core self-evaluations; FWS, future work self salience; CAAS, career adapt-abilities; JSE, job search efficacy; CDATS1, career decision ambiguity tolerance: preference; CDATS2, career decision ambiguity tolerance: tolerance; CDATS3, career decision ambiguity tolerance: aversion; PCBS1, proactive career behavior: career planning; PCBS2, proactive career behavior: skill development; PCBS3, proactive career behavior: career consultation; PCBS4, proactive career behavior: network building; CADS, career decidedness; MVS, my vocational situation” (MVS); SPE1, self-perceived employability: external employability; SPE2, self-perceived employability: internal employability; AS, academic satisfaction.

Table 9 reports the descriptive statistics (means, standard deviations) and reliability coefficients, whereas Figure 8 presents the correlations among the variables. Most measures demonstrated acceptable internal consistency reliability, with Cronbach's alpha coefficients of 0.60 or higher (Nunnally and Bernstein, 1994). However, similar to Study 1, the openness to experience, agreeableness, and conscientiousness subscales of the Big Five showed low internal consistency and were excluded from further analysis. Correlation analyses indicated that most variables in the study were significantly and positively correlated. An exception was the “preference” subscale of career decision ambiguity, which did not exhibit significant association with several variables, including extraversion, neuroticism, future work self, aversion, career decidedness, vocational identity, and academic satisfaction. Neuroticism again demonstrated consistently negative correlations with all other variables.

**Table 9:** Study 2: descriptive statistics and reliabilities (*N* = 323).

No.	Scale	Number of items	*M*	SD	α
1	CFS: willingness to be flexible	6	4.56	0.52	0.69
2	CFS: efficacy in being flexible	3	4.18	0.82	0.60
3	BFI: extraversion	2	2.73	1.05	0.72
4	BFI: neuroticism	2	3.16	0.94	0.62
5	PPS: proactive personality	10	4.82	0.82	0.89
6	CSES: core self-evaluation	12	4.16	0.58	0.86
7	FWS: future work self	5	2.96	0.94	0.93
8	CAAS: career adaptability	24	5.24	0.86	0.95
9	JSE: job search efficacy	8	3.01	0.70	0.86
10	CDATS: preference	4	5.69	0.74	0.79
11	CDATS: tolerance	4	5.07	0.81	0.62
12	CDATS: aversion	4	2.41	1.07	0.76
13	PCBS: career planning	4	3.40	0.81	0.85
14	PCBS: skill development	3	3.64	0.72	0.79
15	PCBS: career consultation	3	3.04	0.90	0.85
16	PCBS: network building	3	3.17	0.87	0.87
17	CADS: career decidedness	6	3.15	0.93	0.89
18	MVS: vocational identity	17	3.17	0.78	0.93
19	SPE: external employability	7	3.32	0.66	0.86
20	SPE: internal employability	4	3.15	0.67	0.78
21	AS: academic satisfaction	7	3.60	0.79	0.91

#### 3.2.4 Extracting the “four As”

As in Study 1, we computed standardized composite scores for each measure and categorized these composites into four distinct groups corresponding to the *adaptive, adaptability, adapting*, and *adaptation* dimensions. Subsequently, we performed separate one-factor CFA for each group.

**Figure 9:**
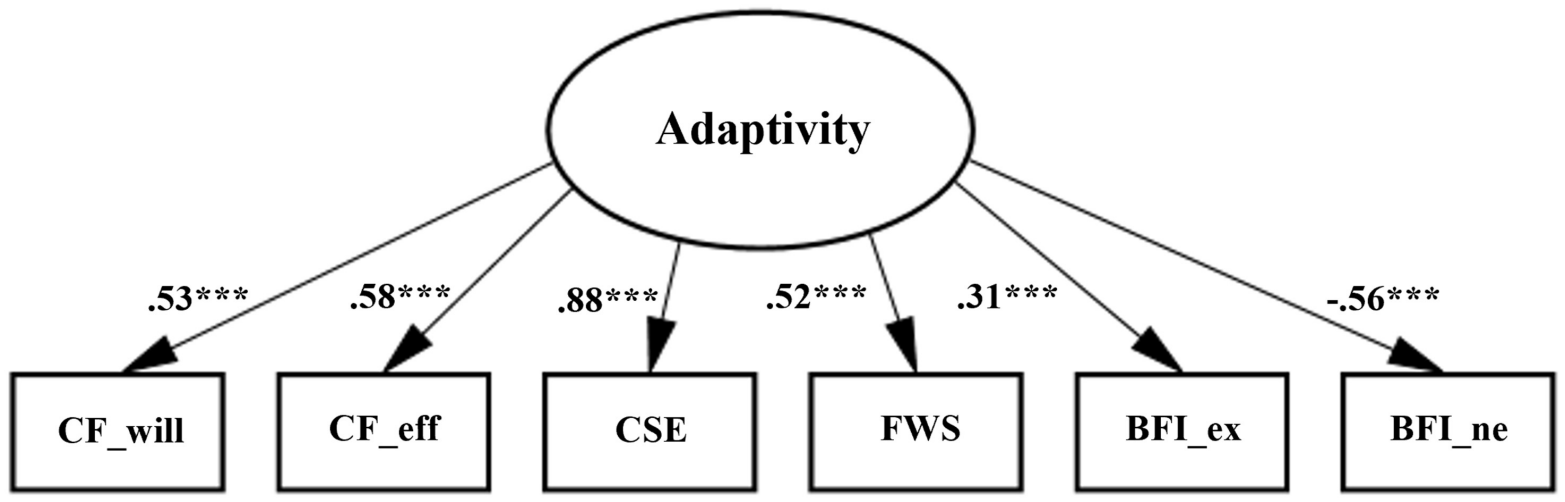
One-factor parceling model of adaptivity (A1): study 2. *N* = 323. Standardized factor loadings are reported in this figure. *** *p* ≤ 0.001. CF_will, cognitive flexibility: willingness to be flexible; CF_eff, cognitive flexibility: efficacy in being flexible; CSE, core self-evaluations; FWS, future work self salience; PP, proactive personality; BFI_ex, big five: extraversion; BFI_ne, big five: neuroticism.

Following the approach of Study 1, we used composite scores of cognitive flexibility, Big Five (extraversion and neuroticism subscales), proactive personality, core self-evaluations, and future work self (total = 7) to examine the factor structure of *adaptivity* (A1). The initial one-factor model demonstrated poor fit (χ^2^ = 91.11, df = 14; CFI = 0.86; SRMR = 0.06). Examining the modification indices revealed that the composite score of proactive personality was affecting the overall model fit, and thus it was removed from the model. The revised one-factor model excluding proactive personality (total number of composites = 6; χ^2^ = 29.83, df = 9; CFI = 0.95; SRMR = 0.04; see Figure 9) met conventional fit thresholds (e.g., Bentler, 1990; Hu and Bentler, 1999).

Unlike Study 1, which assessed the *adaptability* (A2) dimension through Goldman Sachs's (2023) career adapt-abilities inventory alongside Briscoe et al.'s (2006) protean and boundaryless career orientations scales, Study 2 employed only the career adapt-abilities inventory to measure the A2 dimension in university students. We made this adjustment to better align with the student population whose primary focus is exploring career options and developing skills. While the protean and boundaryless career attitudes scales offer valuable insights into career development, they require significant modifications to suit student samples. These scales emphasize navigating multiple career transitions and roles across professional contexts to pursue growth (Wiernik and Kostal, 2019). However, such concepts may not resonate with students who often lack the experience or need for such extensive career shifts. For example, the boundaryless orientation scale includes items like, “I have sought opportunities in the past that allow me to work outside the organization,” which may not be relevant for university students.

**Figure 10:**
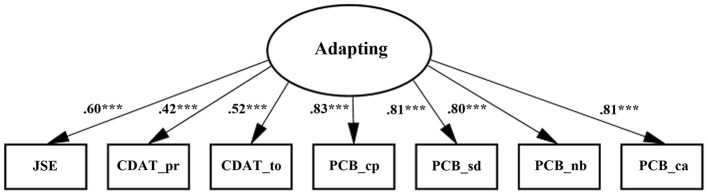
One-factor parceling model of adapting (A3): study 2. *N* = 323. Standardized factor loadings are reported in this figure. *** *p* ≤ 0.001. JSE, job search efficacy; CDAT_pr, career decision ambiguity tolerance: preference; CDAT_to, career decision ambiguity tolerance: tolerance; PCB_cp, proactive career behavior: career planning; PCB_sd, proactive career behavior: skill development; PCB_nb, proactive career behavior: network building; PCB_ca, proactive career behavior: career consultation.

To identify the *adapting* factor (A3), all eight composite scale scores related to proactive career behaviors, job search efficacy, and career decision ambiguity were initially included in the CFA. However, this model did not yield a good fit (χ^2^ = 138.68, df = 20; CFI = 0.89; SRMR = 0.07). Examining modification indices revealed that the model indicated acceptable model fit (χ^2^ = 108.75, df = 14; CFI = 0.91; SRMR = 0.06), after removing the career decision ambiguity tolerance—avoidance subscale (total number of composites = 7; see Figure 10).

**Figure 11:**
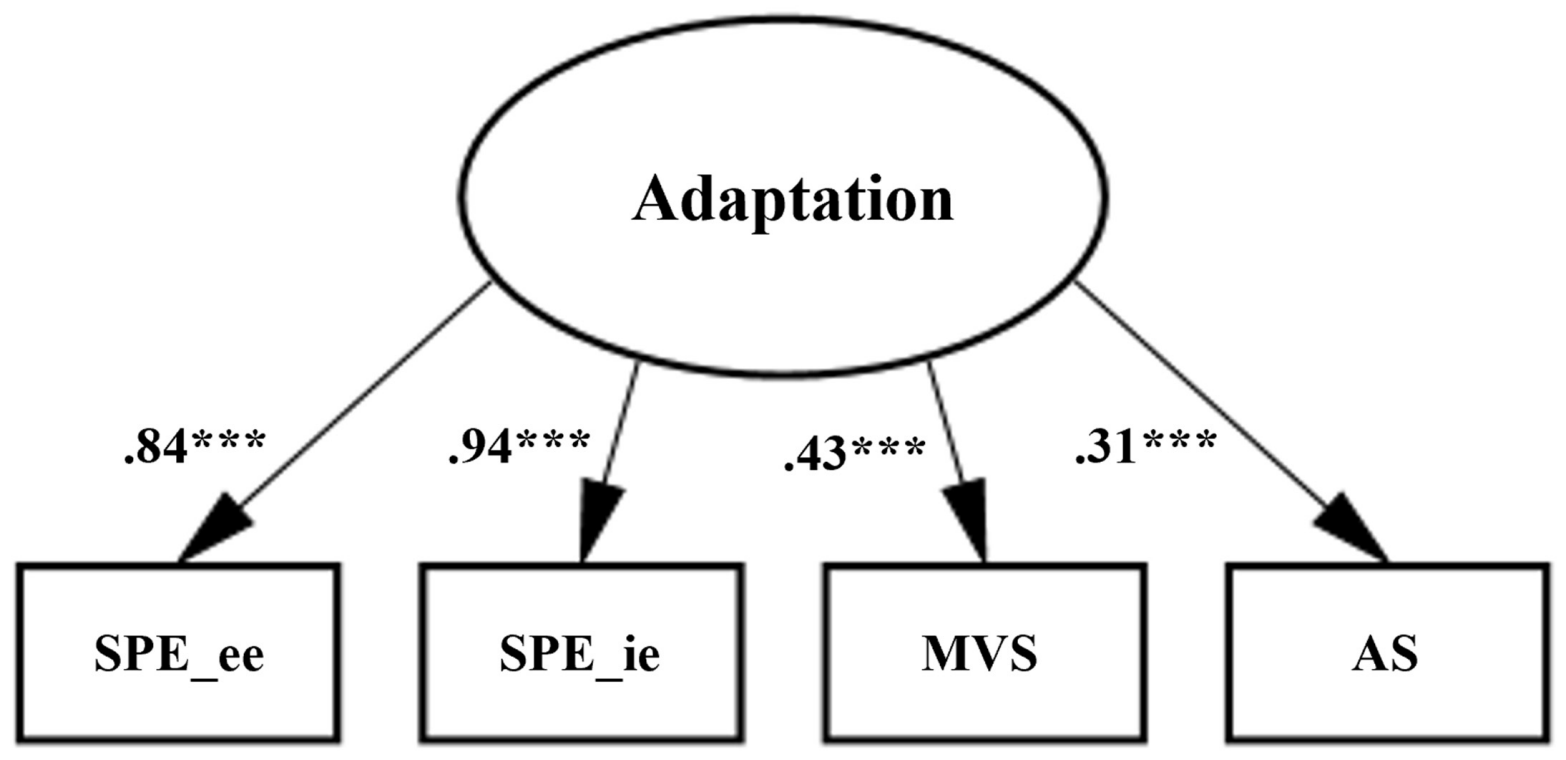
One-factor parceling model of adaptation (A4): study 2. *N* = 323. Standardized factor loadings are reported in this figure. *** *p* ≤ 0.001. SPE_ee, self-perceived employability: external employability; SPE_ie, self-perceived employability: internal employability; MVS, my vocational situation; AS, academic satisfaction.

A model including composite scores from career decidedness, vocational identity, perceived employability, and academic satisfaction measures (total number of composites = 5) resulted in a poor model fit (χ^2^ = 24.07, df = 5; CFI = 0.79; SRMR = 0.11) for the adaptation factor (A4). Removing the career decidedness scale significantly improved the model (total number of composites = 4; see Figure 11). The revised model showed improved fit (χ^2^ = 8.25, df = 2; CFI = 0.98; SRMR = 0.04).

Following the procedures from Study 1, factor scores for each of the “four As” dimensions were computed using regression analysis on the respective final CFA models. These factor scores exhibited moderate positive correlation with each other, ranging from 0.45 to 0.65 (see Table 10 for more details).

**Table 10:** Study 2: correlations among the “four As”.

No.	Factor	1	2	3	4
1	Adaptivity	—			
2	Adaptability	0.45^***^	—		
3	Adapting	0.46^***^	0.56^***^	—	
4	Adaptation	0.49^***^	0.45^***^	0.65^***^	—

*N* = 323. ^***^p ≤ 0.001.

#### 3.2.5 Serial mediation model of the relationships among the “four As”

Similar to Study 1, we compared the partial mediation model against alternative measurement and structural models. As the measurement and partial mediation models were just identified and yielded uninformative fit indices (χ^2^ = 0, df = 0, CFI = 1, SRMR = 0), we relied on AIC and SABIC to assess model adequacy. Detailed fit statistics are provided in Table 11. The partial mediation model again demonstrated superior relative fit compared to models assuming only correlations (AIC = 2,751.80, SABIC = 2,757.39) or full mediation (AIC = 1,969.51, SABIC = 1,972.86).

**Table 11:** Study 2: fit indices of the serial mediation models.

Model	χ^2^	df	χ^2^/df	CFI	SRMR	AIC	SABIC
With CAA as the sole representative of the adaptability dimension							
Correlational path model	0.00	0	N/A	1.00	0.00	2,751.80	2,757.39
Structural model: full mediation	49.80	3	16.60	0.89	0.12	1,969.51	1,972.86
Structural model: partial mediation	0.00	0	N/A	1.00	0.00	1,925.70	1,930.73

*N* = 323. CFI, comparative fit index; SRMR, standardized root mean squared residual; AIC, akaike information criterion; SABIC, sample-size adjusted Bayesian information criterion.

**Figure 12:**
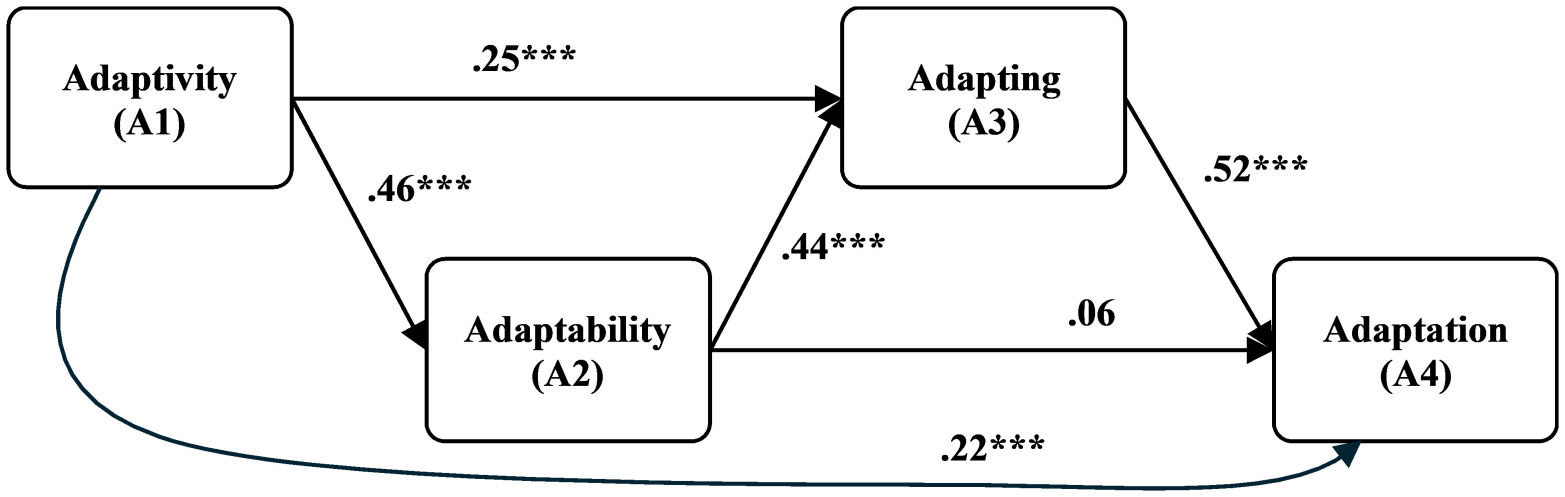
Serial mediation model of the “four As”: study 2. *N* = 323. *** *p* ≤ 0.001.

The serial mediation analysis of the final CCMA model (see Figure 12) revealed that the overall indirect effect of A1 on A4 via A2 and A3 factors was significant (β = 0.11, 95% CI =0.08; 0.16), consistent with the findings of Rudolph et al.'s (2017) meta-analysis on the serial mediation relationships among the adaptivity, adaptability, adapting, and adaptation dimensions. Furthermore, the indirect effects of: (a) A1 on A3 through A2 (β = 0.21, 95% CI =0.15; 0.28), (c) A1 on A4 through A3 (β = 0.13, 95% CI = 0.06; 0.22), and finally (d) A2 on A4 through A3 (β = 0.33, 95% CI = 0.24; 0.44), were significant. Nonetheless, the indirect effect of (b) A1 on A4 through A2 (β = 0.03, 95% CI = −0.03; 0.08) was not significant. For more details about the bootstrap results for indirect effects of the tested mediation model, please refer to Table 12.

**Table 12:** Study 2: bootstrap results for indirect effects.

Indirect effects	β	BCa 95% CI	
		Lower	Upper
A1 → A2 → A3 → A4	0.11^***^	0.08	0.16
A1 → A2 → A4	0.03	−0.03	0.08
A1 → A3 → A4	0.13^***^	0.06	0.22
A1 → A2 → A3	0.21^***^	0.15	0.28
A2 → A3 → A4	0.33^***^	0.24	0.44

*N* = 323. BCa, bias-corrected and accelerated bootstrap interval. ^***^p ≤ 0.001.

## 4 Discussion

### 4.1 Theoretical contributions

The CCMA (Savickas and Porfeli, 2012) highlights the significance of individuals' readiness to make decisions and take action in response to career tasks emerging from ongoing transitions. While the CCMA offers a comprehensive framework that is adaptable to diverse contexts and needs, its flexibility, although advantageous, presents challenges in operationalizing its core concepts, attributing causality, and ultimately developing specific guidance or strategies to nurture individuals' resources and capabilities for successful career development and transitions. This study focuses on assessing the coherence of various measures in delineating a unified construct for representing the adaptivity, adaptability, adapting, and adaptation dimensions (“four As”) of the CCMA. By examining how different measures converge to influence the “four As,” the goal is to enhance the understanding of the underlying mechanisms of the career construction process.

Building on Rudolph et al.'s (2017) meta-analysis of CCMA, our findings identified four common factors that aligned with the conceptualization of the “four As”. These factors, generated from diverse measures, substantiate the essence of the “four As” and underscore their role in shaping career adaptation. The identification of the “four As” across two populations supports the robustness of the CCMA framework in explaining career development across different life stages. While we used nearly identical measures to assess the adaptivity, adapting, and adaptation dimensions, the extracted latent “four As” factors were formed by different scales in each group. This finding suggests that the underlying latent constructs of the “four As” remain consistent even when measured with different indicators, highlighting the flexibility of the CCMA framework in its measurement approach.

In Study 1, we found support for the proposed serial mediation relationships among the “four As” and suggested that these common factors represent meaningful constructs, thereby strengthening the theoretical foundation of CCMA. However, the results of the serial mediation model in Study 2 showed a non-significant relationship between A2 and A4 factors. Specifically, A1 indirectly influenced A4 through the combined effect of A2 and A4, but A2 alone did not appear to significantly mediate the relationship between A1 and A4 for university students. Nonetheless, the CAAS played a significant role for university students. One possible explanation is that the CAAS alone may not fully capture the adaptability dimension of the CCMA in certain contexts. This is indirectly suggested by the serial mediation analysis reported in the working adult sample, where the A2 factor comprising career adapt-abilities, protean career attitudes, and boundaryless mindset scales showed stronger performance compared to models where only CAAS represented the A2 factor. Johnston (2018) discussed the nuanced nature and measurement challenges of the existing career adaptability assessments, specifically the difficulties in distinguishing *adaptability* resources from *adapting* responses. Recent studies (e.g., Haenggli and Hirschi, 2020, 2023) suggested that the four dimensions of the CAAS might not fully encompass the complexities of career adaptability. The inclusion of protean career attitude and boundaryless mindset improved the predictive value of the A2 factor for working adults, suggesting the need to refine career adaptability measures to capture *adaptability* more precisely.

In addition to the measurement challenges associated with A2, another potential explanation for the observed differences between working adults and students lies in the stage of career development and establishment of key adaptation outcomes. For university students, these outcomes (e.g., perceived employability, career decidedness) may not yet be fully realized as they are still navigating early career decisions and developing their professional identity. In contrast, working adults typically have more established career trajectories shaped by concrete outcomes. Theoretical models of career development (e.g., Super and Knasel, 1981; Savickas, 1997; Savickas and Porfeli, 2012) suggest that career adaptability is a dynamic process that evolves over time. For students, it may primarily manifest in exploration, skill development, and testing career choices (Savickas et al., 2018) rather than in the more tangible outcomes seen in working adults. This may partly account for why A2 did not directly associate with A4 for students, as their career outcomes are still in development. This suggests that career interventions for students may focus more on self-awareness, career exploration, and skill development, while interventions for working adults could leverage adaptability to maintain or enhance career success.

We also explored possibilities for more effectively evaluating individual-level career construction. Using factor analysis, we examined which elements play relatively more important roles in constituting the “four As”. For example, the factor solution for the *adaptivity* construct based on the working adults sample showed that while all measures were significantly loaded onto the *adaptivity* factor, cognitive flexibility, proactive personality, and core self-evaluation measures exhibited relatively stronger loading coefficients compared to other measures. These findings offer valuable guidance for future research seeking to refine assessments and streamline the measurement of the career construction process.

### 4.2 Practical implications

The observed differences between working professionals and university students suggest that career adaptability functions in distinct ways across career stages. Career development programs and interventions should therefore tailor their content to reflect life-stage-specific needs and capacities (Savickas, 2005). Designing interventions that recognize how adaptability resources translate differently into outcomes can enhance their relevance and impact.

For example, the weaker adaptability–adaptation link among students underscores the transitional nature of early career stages, where self-regulatory capacities may not immediately yield concrete outcomes. Unlike working adults, students often lack the opportunity or agency to enact career behaviors in organizational settings (Savickas et al., 2018). As such, recent graduates may benefit from targeted support that helps them bridge the gap between adaptability and real-world career outcomes, such as structured mentoring, experiential learning, or transition coaching. By aligning intervention strategies with career stage, practitioners can more effectively foster career agency and long-term employability across diverse populations.

### 4.3 Limitations and future research

While we focused on a targeted subset of constructs within the Career Construction Model of Adaptation (CCMA), we selected these variables based on their theoretical significance, empirical support, and frequent application in existing literature. This approach allowed us to ensure construct validity, reliability, and comparability with prior research. Nonetheless, future studies could expand on our work by incorporating additional CCMA dimensions to capture a more comprehensive representation of the framework.

The cross-sectional design of the present study limits causal inference. While the observed associations are consistent with theoretical expectations, the temporal ordering of the CCMA dimensions cannot be definitively established. Longitudinal research would allow for a more rigorous examination of how career adaptation processes unfold over time and how these dimensions interact dynamically across different career stages.

Although largely consistent instruments were used across samples, some differences in instrument selection were introduced to ensure relevance for working adults and university students respectively. While this approach better reflects the lived contexts of each population, it may limit direct comparability across samples. Future research could address this by replicating the present study within each population using a comparable set of measures, allowing for cleaner within-sample comparisons over time.

More broadly, the observed difference between samples highlights the challenge of replicating the full serial mediation model across populations with different career development stages. In this context, available measures may not equally capture the essence of each of the “four As”. As demonstrated in Study 1, CAAS alone may be insufficient to represent A2. Similarly, the measures available for A4 in the student sample, while appropriate in scope, may not yet fully reflect the career outcomes such as perceived employability (Rodrigues et al., 2019) and career decidedness (e.g., Lounsbury et al., 2005), which are likely less consolidated at this life stage (Gianakos, 1996; Super and Knasel, 1981).

We used self-report measures to assess cognitive ability, career behaviors, and outcomes. This approach is well-established in career research and suitable for capturing individuals' subjective experiences (Cooper et al., 2020). Although self-report methods offer important insights, future research could complement them with alternative assessment tools to enrich data triangulation. For instance, studies assessing cognitive flexibility may benefit from combining self-reports with performance-based neuropsychological tasks, such as the CANTAB Intra/Extra Dimensional Set Shifting Task (Robbins et al., 1998) or the Wisconsin Card Sorting Task (Grant and Berg, 1948), to provide a more holistic understanding of underlying cognitive processes. Nevertheless, while incorporating task-based cognitive measures could further advance our understanding of how cognitive capacities influence career development, researchers should be mindful of the conceptual distinctions between self-reported measures and neuropsychological tasks. Self-reports capture psychological flexibility and align more closely with the CCMA's operationalization of *adaptivity* as a trait-like readiness to change (Savickas, 2002, 2012), whereas neuropsychological tasks assess attentional switching in response to external feedback (Diamond, 2006, 2013) and reflect a distinct facet of cognitive flexibility (Hohl and Dolcos, 2024; Howlett et al., 2021; Johnco et al., 2014). Each type therefore serves a distinct inferential purpose.

Relating to self-report measures, we also acknowledge that the high inter-correlations observed among the ‘four As' in Study 1 (≥0.76) may raise concerns about conceptual overlap. However, these values are consistent with the theoretically integrated nature of the CCMA, wherein each dimension functions as a precursor to the next (Savickas, 2002; Savickas and Porfeli, 2012), and elevated inter-correlations have been noted as characteristic of CCMA-related constructs in prior research (Leung et al., 2022; Rudolph et al., 2017). Importantly, this pattern was not observed in Study 2, where inter-correlations were more moderate (*r* = 0.45–0.65). The mixed pattern of positive, negative, and non-significant associations observed across both studies also suggests that the constructs remain empirically distinguishable despite their conceptual relatedness.

## 5 Conclusion

The CCMA framework highlights the significance of individuals' readiness to make decisions and take action in response to career tasks emerging from ongoing transitions. While the CCMA provides a comprehensive and adaptable framework for diverse contexts and needs, its very flexibility poses challenges in consistently operationalizing core concepts, establishing causal relationships, and developing targeted strategies to strengthen individuals' resources and capabilities for effective career development and transition.

This paper aimed to evaluate the coherence of various measures in defining a unified construct for the dimensions of *adaptivity, adaptability, adapting*, and *adaptation* (“four As”) within the CCMA framework. By systematically analyzing how different measures converge to represent the “four As” across two distinct populations, namely working professionals and university students, this research offers new insights into the underlying mechanisms of the career construction process.

## Data Availability

The raw data supporting the conclusions of this article will be made available by the authors, without undue reservation.
