# Supplementary material for: A psychometric examination of the career construction model of adaptation in working adults and students
Source: Front Psychol. 2026 Jul 27;17:1799936. doi: 10.3389/fpsyg.2026.1799936 (PMC13454876; doi:10.3389/fpsyg.2026.1799936)
Supplement: Supplementary file 1 [file Supplementary_file_1.docx]

# Supplementary Material

## **A List of measures**

All measures used in this paper are well established and widely recognized, with a high number of citations supporting their validity and reliability.

**Adaptivity**

***Cognitive Flexibility***. Martin and Rubin’s (1995) Cognitive Flexibility Scale (CFS) is a self‑reported assessment designed to measure individuals' cognitive flexibility. This scale comprises 12 items that examine: (a) awareness of available alternatives in one's surroundings, and (b) efficacy in being flexible. Participants rate each item on a 7‑point Likert scale (1 – Strongly disagree; 7 – Strongly agree). An example item from the CFS is, for instance, “I can communicate an idea in many different ways.”

***Big Five Personality***. Rammstedt and John’s (2007) 10‑item version of the Big Five Inventory (BFI‑10) aims to assess an individual's personality across the Big Five domains: extraversion, neuroticism, conscientiousness, agreeableness, and openness. The scale is measured using a 5‑point Likert scale (1 – Disagree strongly; 5 – Agree strongly). A sample item from the BFI‑10 is, for instance, “I see myself as someone who is outgoing and sociable.”

***Proactive Personality***. Seibert et al.'s (1999) 10‑item shortened Proactive Personality Scale (PPS) is designed to measure a personal disposition toward proactive behavior, i.e., taking initiative to make changes in their environments. The scale was rated using a 7‑point Likert scale (1 – Strongly disagree; 7 – Strongly agree). A sample item from the PPS is, for instance, “I am constantly on the lookout for new ways to improve my life.”

***Core Self-Evaluations***. Judge et al.’s (2003) 12‑item Core Self‑Evaluations Scale (CSES) was developed to measure four core personality traits: self‑esteem, generalized self‑efficacy, neuroticism, and locus of control. The scale is measured using a 5‑point Likert scale (1 – Strongly disagree; 5 – Strongly agree). A sample item from the CSES is, for instance, “I am confident I get the success I deserve in life.”

***Future Orientation***. Strauss et al.’s (2012) 5‑item Future Work Self Salience (FWS) aims to engage participants in envisioning their professional selves in the future. The scale is measured using a 5‑point Likert scale (1 – Strongly disagree; 5 – Strongly agree). A sample item from the FWS is, for instance, “I am very clear about who and what I want to become in my future work.”

**Adaptability**

***Career Adaptability***. Savickas & Porfeli’s (2012) Career Adapt‑Abilities Scale (CAAS) – international version 2.0 comprises 24 items with four facets of career adaptability: concern, control, curiosity, and confidence. The scale is measured using a 5‑point Likert scale (1 – Not strong; 5 – Strongest). A sample item from the CAAS 2.0 is, for instance, “Planning how to achieve my goals.”

****Protean Career Attitude***. Briscoe & Hall's (2006) Protean Career Attitude Scale (PCAS) was designed to measure two aspects of the protean career attitude: self‑directed attitude and value-driven attitude. The scale contains 14 items, rated on a 5‑point Likert scale (1 – To little or no extent; 5 – To a great extent). A sample item from the PCAS is, for instance, “I navigate my own career, based on my personal priorities, as opposed to my employer’s priorities.”

****Boundaryless Career Attitude***. Briscoe & Hall's (2006) Boundaryless Career Attitude Scale (BCAS) was designed to measure two of the boundaryless career attitudes: boundaryless mindset and organizational mobility preference. The scale contains 13 items, rated on a 5‑point Likert scale (1 – To little or no extent; 5 – To a great extent). A sample item from the BCAS is, for instance, “I enjoy job assignments that require me to work outside of the organization.”

**Adapting**

***Proactive Career Behavior***. Strauss et al.’s (2012) 13‑item Proactive Career Behavior Scale (PCBS) examines four types of proactive career behaviors: career planning, proactive skill development, career consultation, and network building. The scale is rated on a 5‑point Likert scale (1 – Strongly disagree; 5 – Strong agree). A sample item from the PCBS is, for instance, “I develop knowledge and skills in tasks critical to my future work life.”

***Job Search Efficacy***. Eleven items from Wanberg et al.’s (2010) “Getting Ready for Your Next Job” Inventory that measuring job search efficacy were included in Study 1 to assess individuals’ self‑rated skills for effectively managing specific job search tasks. The items were rated using a 5‑point Likert Scale (1 – Not confident at all; 5 – Extremely confident). A sample item from the JSE is, for instance, “How confident do you feel about being able to do a good job of changing your resume to fit specific jobs?”

***Career Decision***‑***Making Efficacy***. Xu et al.’s (2015) Career Decision Ambiguity Tolerance Scale (CDATS) was developed to assess how individuals evaluate and respond to ambiguous information during the decision‑making process within a career context. The scale comprised eighteen items tapping into three factors (preference, tolerance, aversion) and was rated using a 7‑point Likert scale (1 – Strongly disagree; 7 – Strongly agree). A sample item from the CDATS is, for instance, “I enjoy tackling complex career decision‑making tasks.”

**Adaptation**

***Career Decidedness***. Lounsbury et al.’s (2005) 6‑item Career Decidedness Scale (CADS) was developed to assess individuals’ determination regarding their career choice. The scale was rated using a 5‑point Likert scale (1 – Strongly disagree; 5 – Strong agree). A sample item from the CADS is, for instance, “I am sure about what I eventually want to do for a living.”

***Vocational Identity***. Holland et al.'s (1980) “My Vocational Situation” (MVS) questionnaire was developed to examine identity diffusion in the career context, assuming that the lower individuals establish vocational identity, the more barriers they perceive to making career decisions and achieving their goals. The scale consists of 18 items and was rated using a 5‑point Likert scale (1 – Not true; 5 – True). A sample item from the MVS is, for instance, “I can’t understand how some people can be so set about what they want to do.”

***Perceived Employability***. Rothwell & Arnold’s (2005) 11‑item Self‑Perceived Employability Scale (SPE) was developed to assess individuals’ perceptions of their employability within and outside of their current organization, taking into account both personal and occupational attributes. The scale was rated using a 5‑point Likert scale (1 – Strongly disagree; 5 – Strongly agree). A sample item from the SPE is, for instance, “Even if there was downsizing in this organization I am confident that I would be retained.”

****Work Engagement***. Avallone et al.’s (2007) 10‑item Work Self‑Efficacy Scale (WSES) was developed to assess individuals’ capability to commit themselves to their jobs and manage their professional networks. The scale was rated using a 5‑point Likert scale (1 – Not well at all; 5 – Extremely well). A sample item from the WSES is, for instance, “How well can you respect schedules and working deadlines?”

****Turnover Intention***. Bothma & Roodt’s (2013) Turnover Intention Scale (TIS) was employed to assess individuals’ intention to leave their current organization. The abbreviated scale, known as TIS‑6, comprises six items and was rated using a 5‑point Likert scale (1 – Rarely; 5 – Always). A sample item from the TIS‑6 is, for instance, “How likely are you to accept another job at the same compensation level should it be offered to you?”

**^†^*Academic Satisfaction***. Lent et al.’s (2005) 7‑item Academic Satisfaction (AS) scale assesses participants’ satisfaction with various aspects of their academic experience. Participants rate each of the 7 items on a 5‑point scale, where 1 represents “strongly disagree” and 5 represents “strongly agree.” A sample item from the AS is, for example, “I enjoy the level of intellectual stimulation in my courses.”

*Note*. * Scales included only in Study 1; † Scales included only in Study 2./
